# A novel approach to assessing the antioxidant and anti-diabetic potential of synthesized calcium carbonate nanoparticles using various extracts of Ailanthus altissima

**DOI:** 10.3389/fchem.2024.1345950

**Published:** 2024-06-03

**Authors:** Noreen Samad, Umer Ejaz, Saba Kousar, Aamal A. Al-Mutairi, Arslan Khalid, Zeemal Seemab Amin, Shahzad Bashir, Sami A. Al-Hussain, Ali Irfan, Magdi E. A. Zaki

**Affiliations:** ^1^ Department of Biochemistry, Faculty of Science, Bahauddin Zakariya University, Multan, Pakistan; ^2^ Department of Chemistry, College of Science, Imam Mohammad Ibn Saud Islamic University (IMSIU), Riyadh, Saudi Arabia; ^3^ Department of Biochemistry, Faculty of Applied Sciences, Minhaj University Lahore, Lahore, Pakistan; ^4^ Department of Chemistry, Government College University Faisalabad, Faisalabad, Pakistan

**Keywords:** calcium carbonate nanoparticles, green synthesis, antioxidants, antidiabetic, plant extract, molecular docking, ADMET studies

## Abstract

Calcium carbonate nanoparticles (CaCO_3_) have been found to exhibit unique properties that show their potential to be used in various therapies. Green synthesis of CaCO_3_ has been progressively gaining ac-ceptance due to its cost-effectiveness and energy-efficient nature. In the current study, different extracts of *Ailanthus altissima* were used to synthesize the calcium carbonate nanoparticles the synthesis and characterization of CCNPs were confirmed by using Fourier Transform Infra-Red spectroscopy, UV-Vis spectroscopy, and Scanning Electron Microscopy (SEM). The antioxidant activities (hydrogen peroxide, phosphomolydbenum, and ferric reducing) of calcium carbonate nanoparticles were affirmed by a good range of percentages of inhibition against free radical scavenging. The antidebate assays of CCNPs were observed by *in-vitro* and *in silico* approaches in a range at various concentrations while maximum inhibition occurred. In conclusion, the current study depicted that conjugated CaCO_3_ with *A. altissima* has a good potential to cure oxidative stress and Type II diabetes and could be used in the future as biogenic nanomedicine for the treatment of other metabolic diseases.

## 1 Introduction

Currently, nanotechnology is being used in many aspects of everyday existence including medicine, drug delivery, electronics, cosmetics, and food processing (Garg et al., 2021). Nanoparticles (NPs), as per the International Organization for Standardization (ISO), an international consortium of national standards organizations, are characterized as nano-objects whose physical dimensions exclusively fall within the nanoscale range, with no notable discrepancy in length between their longest and shortest axes (Nam and Luong, 2019). Three sizes of NPs can be classified as the nanoscale: larger than 500 nm, 100 nm–500 nm, and 1 nm to 100 nm. In general, the nanoscale ranges from one to 100 nm. Depending on their dimensions and the distribution of those dimensions, NPs may manifest size-dependent intensive characteristics (Nam and Luong, 2019). Most often, inorganic NPs are investigated for their ranostic potential, i.e., their ability to combine diagnosis and treatment. Inorganic NPs used in biomedical applications comprise pure metals (particularly plasmonic NPs, i.e., Ag and Au), metal oxides, and calcium phosphates (Asad et al., 2022). Due to their extensive utility across various industrial processes, metal oxide-based nanomaterials have made significant strides in their development. These include catalytic applications, plasmonics, and fuel production. Researchers have been interested in calcium carbonate NPs (CaCO_3_NPs) because of their unique chemical and physical characteristics (Fadia et al., 2021). Numerous NPs have been fabricated through the utilization of fruit peels, pulps, and various plant components as agents for reduction. In bones and teeth, calcium derivatives play an important role. The predominant composition of bone tissue comprises a composite material with calcium serving as its principal constituent (Garg et al., 2021). Calcium carbonate (CaCO_3_) is used to administer drugs precisely, as well as to encapsulate various pharmaceuticals, including bioactive proteins, because it is readily available and biodegradable. The micro- and NPs of calcium carbonate are widely recognized as chemically inert substances biosensors, drug delivery, filler materials, paint, sealants, and adhesives use them for biosensing, drug delivery, and filler materials. (Fadia et al., 2021; DAmora et al., 2020; Supit and Shaikh, 2014). The properties of oxides are outstanding from a structural, biocompatible, and mechanical standpoints (Shadianlou et al., 2022; Bapat et al., 2022). The bacteriostatic properties of metal oxide NPs are generally attributed to the release of ions (s) and the generation of reactive oxygen species. According to several studies, NPs have several physicochemical effects depending on their size shape, and type of chemical modification, which has a significant impact on their antibacterial properties in green nanomaterials such as CaCO_3_. In addition to improving the intracellular penetration and retention time of antibiotics, NPs could also be a potential delivery system for antimicrobial agents. Recently, calcium carbonate nanoparticles have been largely integrated with imaging contrast and therapeutic agents for various imaging and therapeutic approaches (Zhao et al., 2022).

Diabetes is a chronic disease caused by a combination of hereditary and environmental factors leading to abnormally high blood glucose levels. In both developed and developing countries, it is a major health concern. α-Amylase and α-glucosidase (Tetrameric 11B-HSD1) digest the carbohydrates and increase the postprandial glucose level in diabetic patients (Poovitha and Parani, 2016). There are many therapies available to treat diabetes, but they do not cure the disease completely and have several adverse effects (Jini and Sharmila, 2020). Advanced glycation end-products (AGEs) resulting from the process of protein glycation have been linked to diabetic complications. Multiple research endeavors have demonstrated a strong correlation between the accrual of AGEs within tissues and the onset of various ailments, encompassing diabetic complications, Alzheimer’s disease, renal impairment, and cardiovascular disorders (Zhao et al., 2021). Nanotechnology has enabled the synthesis of NPs from natural materials that are effective inhibitors of “glucosidase” and “amylase” enzymes in treating diabetes (Khan et al., 2023). Furthermore, computational analysis simplifies the process of identifying the conventional uses of drugs against diabaties. This type of analysis can also be used to identify potential uses for drugs that have not yet been discovered. Computational analysis can also be used to predict how new drugs interact with the body, allowing for more efficient drug development (Dige et al., 2020).

*Ailanthus altissima (Mill.)* Swingle belongs to Simaroubaceae and is a deciduous tree indigenous to the northern and central regions of China, Japan, Taiwan, and Vietnam, it is more commonly recognized by its vernacular name, the “tree of heaven” (Albouchi et al., 2013). The ornamental characteristics of this species have led to its introduction and naturalization in many countries around the world. The plant contains important chemical constituents such as -pinene, -pinene, and -terpinene which are utilized for addressing a wide variety of medical disorders. (Shabbir Awan et al., 2023). This tree holds a significant role due to its wide-ranging utility in indigenous healthcare, where it is employed for the management of amoebic dysentery, gastric ailments, and colds. Extensive research has documented its various pharmacological properties, including antioxidant, antifungal, antimalarial, antituberculosis, antiviral, insecticidal, cytotoxic, anti-inflammatory, antiasthmatic, antiproliferative, and phosphodiesterase inhibitory activities (Albouchi et al., 2013). The phytochemical constituents within the plant extract serve a dual purpose, serving as both stabilizing and reducing agents in the process of nanoparticle synthesis. Utilizing plant extract for green synthesis of NPs offers distinct advantages compared to alternative methodologies due to its, one-step procedure, inherent simplicity, environmentally conscientious nature, and cost-efficiency. While the scientific literature has extensively documented the chemical synthesis of calcium carbonate, scant information is accessible about the environmentally friendly, green synthesis of this valuable compound in nanoparticle form (Garg et al., 2021). First and foremost to evaluate the bioactive potential of calcium carbonate nanoparticles of different extracts of *A. altissima*.

The goal of this study was to determine the biosynthesis of CaCO_3_ -NPs extracted from various fractions of *A. altissima*. CaCO_3_-NPs can be produced by plant extracts of different fractions and are a fast and economical alternative to synthetic methods. CaCO_3_-NPs have been reviewed for their effectiveness in antidiabetic and anti-free radical scavengers, and anti-glycation, besides their potential for accessing further biological functions.

## 2 Materials and methods

### 2.1 Collection and identification of plant

The *A. altissima* were purchased from a nursery in Multan*, Pakistan.* Plant identification was made possible by the Department of Botany at Bahauddin Zakariya University, Pakistan. There was a voucher number of *A. altissima* [kew-2626,815 (2,734)] associated with it.

### 2.2 Chemicals

Sodium carbonate (Na_2_CO_3_), ethanol (CH_2_OH), Methanol (CHOH), Calcium chloride (CaCl_2_), hydrogen peroxide (H_2_O_2_), Vitamin C, sulfuric acid (H_2_SO_4_), Ammonium molybdate ([NH_4_]2MoO_4_, Sodium phosphate buffer (Na_3_PO_4_), Potassium ferricyanide (C_6_N_6_FeK_3_), α-amylase (C_9_H_14_N_4_O_3_), Tri-chloroacetic acid (C_2_HCl_3_O_2_), Ferric chloride (FeCl_3_). All the used chemicals were analytical grade and highly provided and purchased from Sigma Aldrich United States.

### 2.3 Preparation of extracts of bark of *Ailanthus altissima*

The fresh bark of *A. altissima* was collected and washed with tap water before being washed with distilled water under running water until there were no impurities left. In a 250 mL flask, 10 g of powdered bark was supplemented with 100 mL of deionized water. An electric stirrer with a speed of 500 rpm was used to stir the powder into methanol and ethanol and water mixture for 1 hour at room temperature. To filter it, Whatman no.1 filter paper was used. The crude extracted material was stored at 4°C for future use. To synthesize CaCO_3_, extracts were refrigerated (Ghasemzadeh et al., 2015; Aritonang et al., 2019).

### 2.4 Synthesis of calcium carbonate nanoparticles

The green synthesis of CaCO_3_ was carried out by a slight modification method by Garg, Kumari (Garg et al., 2021). Each flask was filled with 25 mL of methanol and ethanol aqueous crude extract separately. Following this 50 mL of 5 × 10^−2^ molL-1 CaCl2 aqueous solution was gently added in each beaker. The pH was then stabilized at 8.5 by adding one molL-1 of ammonia aqueous solution. Using a magnetic stirrer, the flasks were stirred for 2 hours. A brown color change was observed in samples after the addition of Na2CO3. The flasks were then plugged with cotton and incubated for 2–3 days at 27°C. The precipitates were separated by centrifugation and appeared at the bottom of the flasks. Distilled water, ethanol, and chloroform were used to wash the precipitates. Precipitates become air-dried after washing. The air-dried powder was used for characterization and other biological activities (Garg et al., 2021).

#### 2.4.1 Characterization of biosynthesized CaCO_3_

At standard room temperature, optical absorption spectra were acquired of CaCO_3_-NPs double-beam V-630 spectrophotometer operating in the 100–700 nm wavelength range was used for the analysis (Shimadzu UV-2500PC Series, Kyoto, Japan). Meanwhile, Characterization f potent bioactive groups linked to the surface of nanoparticles was performed by using Perkin-Elmer spectrometer system (Perkin Elmer United States ASTM E 2412), with a resolution of 4 cm^−1^ within a peak range of 450–4,000 cm^−1^ (Abdelhafez et al., 2020). The surface characteristics of the specimens were assessed using a Scanning Electron Microscope (SEM, Carl Zeiss: EVO40) operating at 20 kV following the application of a thin platinum layer via sputter coating (Sputter Coater: POLARONSC7640). After that, analysis was performed using an EDX (EDX, model 7,353, England), detector attached to the SEM which was directly interfaced with the VPSEM (Islam et al., 2012; Ghadami Jadval Ghadam and Idrees, 2013).

### 2.5 Biological activities

#### 2.5.1 Hydrogen peroxide radical scavenging activity

The assessment of hydrogen peroxide scavenging activity (HPSA) in *A. altissima* extracts, CaCO_3_, and Vitamin C was conducted following the method outlined by (Keshari, 2021) with minor adjustments. Specifically, 2 mL of *A. altissima* extract in ethanol, methanol, and aqueous solutions (concentrations ranging from 50 μg/mL to 250 μg/mL, all in 50 mM phosphate buffer at pH 7.4) were combined with 0.3 mL of 50 mM phosphate buffer (pH 7.4) and 0.6 µL of a 2 mM H2O2 solution in 50 mM phosphate buffer. The solution was subjected to a 10-min incubation period, during which absorbance measurements were taken at a wavelength of 230 nm (Keshari, 2021). This experimental protocol was replicated for both CaCO_3_ and vitamin C. Subsequently, absorbance readings at 230 nm were obtained, and the findings were reported as a percentage of inhibition.

#### 2.5.2 Phosphomolybdenum assay

Phosphomolybdenum assay was performed according to the (Thirugnanasambandam et al., 2016). The reaction mixture was meticulously prepared by combining equal volumes of phosphate buffer, sulfuric acid (H2SO4), and ammonium molybdate, ensuring thorough mixing. Subsequently, 1 mL of each respective extract and corresponding NPs were dispensed into individual test tubes. 3 mL of the prepared reaction mixture was added to these test tubes. The test tubes were securely sealed with aluminum foil and incubated at a temperature of 95°C for 90 min. Following incubation, each test solution was allowed to cool to room temperature, and their respective absorbance readings were recorded at 695 nm using a blank reference. It is important to note that vitamin C was employed as a control in this experimental procedure. An identical protocol was adhered to for the preparation of CaCO_3_ and vitamin C.

#### 2.5.3 Ferric-reducing anti-oxidant power assay

With slightly modified changes ferric reducing anti-oxidant power assay was performed by (Subramanian et al., 2013). All test tubes were then filled with 2.5 mL of sodium phosphate buffer, 2.5 mL of potassium ferricyanide solution, and 2.5 mL of different extracts and their respective NPs. After thorough vortex mixing, thereaction mixtures in all test tubes were incubated at 50°C for 20 min. Following the addition of 2.5 mL of trichloroacetic acid to each tube, they were centrifuged at 3,000 rpm for 10 min. Subsequently, 2.5 mL of supernatant was mixed with 0.5 mL of ferric chloride and 2.5 mL of deionized water to create a colored solution. The absorbance of this solution was measured at 700 nm using a spectrophotometer against a blank. Ascorbic acid was employed as a reference standard.

#### 2.5.4 α-amylase inhibition assay

In a controlled laboratory environment, 250 µL of starch and α-amylase solution was added to all test tubes, followed by 250 µL of extracts and their corresponding NPs. After a 3-min incubation at 20°C, 500 µL of dinitrosalicylic acid was introduced to halt the enzymatic reaction. The mixture was briefly heated in boiling water, and 250 µL of α-amylase solution was promptly added. After a 15-min heating and subsequent cooling to room temperature, the total volume was adjusted to 6,000 µL by adding 4,500 µL of distilled water. Thorough homogenization was achieved using a vortex. It is worth noting that the blank reference sample contained starch and enzymes but lacked any extracts or NPs (Yarrappagaari et al., 2020). The assessment of *α*-amylase enzymatic activity was conducted at a wavelength of 540 nm utilizing a spectrophotometer. This measurement relied on the quantification of the absorbance resulting from the reduction of dinitrosalicylic acid, a reaction product indicative of maltose formation. The percentage inhibition was measured by using the following equation:

$$\text{Inhibition }\left(\%\right)=\left(A1\;–\;A2\right)\;/\;A1\times 100$$

A1 indicates the absorbance of blank while A2 indicates the absorbance of samples.

#### 2.5.5 α-glucosidase inhibition assay

The method previously described was used to test the inhibitory activity of α-glucosidase. In summary, 10 μL of 0.25 U/mL α-glucosidase (Sigma-Aldrich, USA), 50 μL of 0.1 M potassium phosphate buffer (pH 6.8), and 20 μL of the 250 μL of extracts and their corresponding NPs or the α-glucosidase inhibitor acarbose (Fluka, USA) were combined and incubated for 10 minutes at 37 °C. Subsequently, 30 minutes were spent incubating 10 μL of 5 mM pnitrophenyl-α-D-glucopyranoside (PNPG). A volume of 50 μL of 0.1 M Na2CO3 was injected to stop the reaction.

Optical measurements of the absorbance at 405 nm were made with a spectrophotometer (Doan et al., 2018).

In-silico study of NPs against type 2 diabetes.

##### 2.5.5.1 Ligand selection

A literature review of *A. altissima* revealed 20 bioactive flavonoids. ChemDraw version 2023 software was used to draw the structure and convert into (SMILES) structure. In addition, the PDB structure is obtained from https://cactus.nci.nih.gov/translate/(Mohammed et al., 2022).

##### 2.5.5.2 Selection of target protein

In the scientific literature, a susceptibility protein has been identified. The three-dimensional structures of these target proteins, with accession codes Alpha-amylase (1b2y) and Tetrameric 11b-HSD (1xu7) have been deposited and are accessible in the Protein Data Bank (PDB)repository.

##### 2.5.5.3 Docking studies

The PyRx software was employed for the docking simulations involving two protein structures, Alpha-amylase 1b2y and Tetrameric 11b-HSD 1xu7, in conjunction with 20 flavonoid ligands derived from *A. altissima.* The ligands were subjected to preparation using PyRx’s Open Babel functionality, while the target protein underwent preparation using Discovery Studio 2022. Furthermore, the analysis incorporated the utilization of Discovery Studio 2022 as an integral component (Afzal et al., 2023).

##### 2.5.5.4 Molecular dynamics simulations

The MDS complexes underwent a comprehensive analysis involving deformability assessment, B-factor analysis, covariance analysis, and root-mean-square fluctuation evaluation to identify any residues that may exhibit instability or deformation following the coarse-grained Molecular Dynamics Simulations (MDS). Deformability, B-factor, and covariance analyses were conducted utilizing the iMODS software (Qazi et al., 2021)

##### 2.5.5.5 Pharmacokinetic properties

Using Swiss ADME, drug-likeness, small molecule pharmacokinetics, and medicinal chemistry properties can be evaluated. PK-CSM4 and admetSAR5 are two free web-based applications for pharmaco kinetics and ADME. The Swiss ADME system offers a unique combination of proficient methods, such as boiled eggs, as well as non-exhaustive input methods, multiple molecule computations, and saving, displaying, and sharing results either on a local or worldwide scale. In the concluding stage, the integration of Swiss ADME is incorporated into the framework of Swiss Drug Design (Awadelkareem et al., 2022).

## 3 Results

### 3.1 Characterization techniques

The production of CaCO_3_ was confirmed through UV-Vis spectroscopy, SEM, EDX, and FTIR.

#### 3.1.1 UV-vis spectrophotometry analysis

**FIGURE 1:**
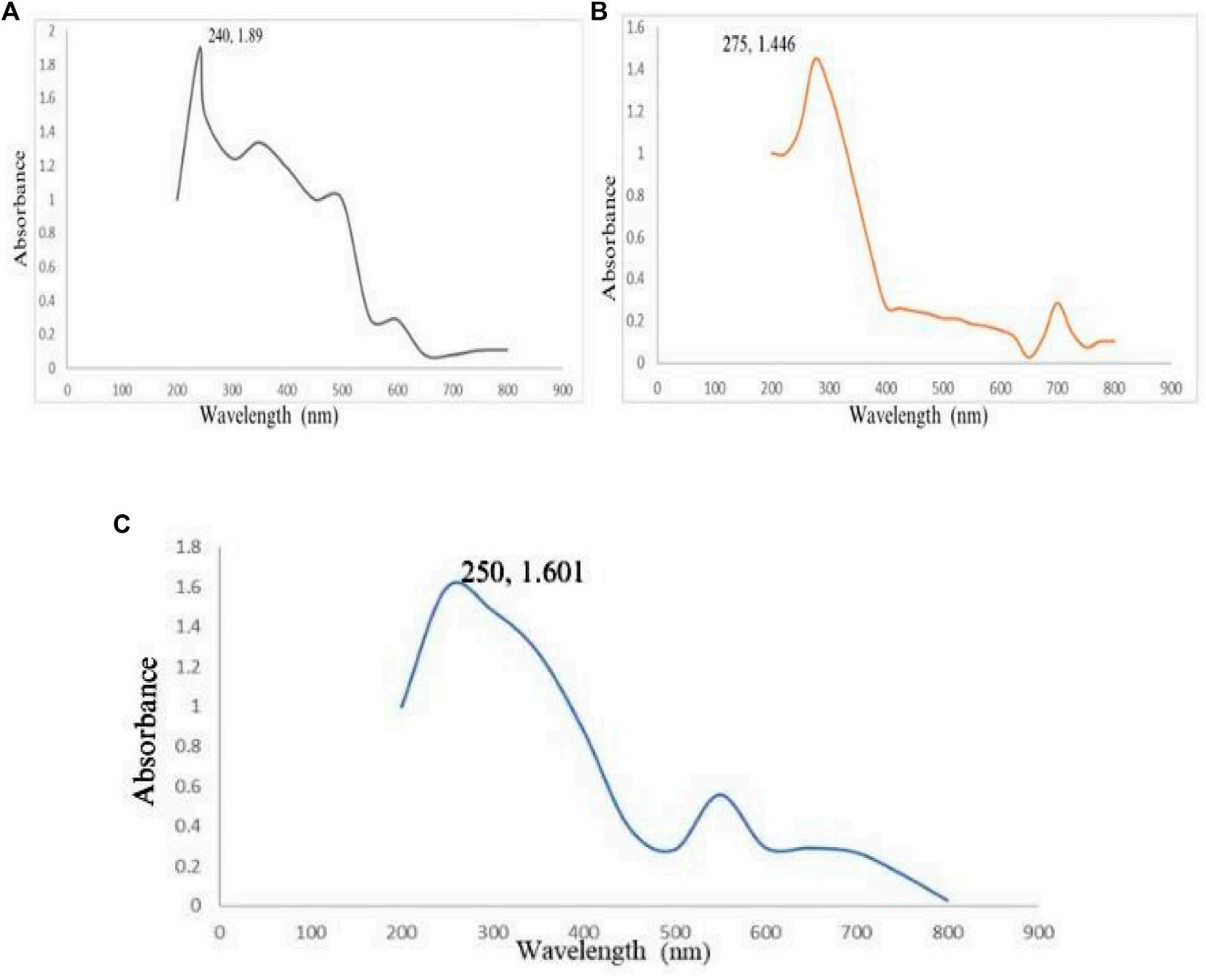
UV-visible spectra of CaCO_3_ of different extracts of *Ailanthus altissima* Figure **(A)** shows ethanol while **(B)** and **(C)** show methanol and aqueous.

By observing the color change during the reaction, it was confirmed that CaCO_3_-NPs were synthesized from *A. altissima* bark extracts. Following this, UV-visible spectrophotometry was employed to validate the synthesis of CaCO_3_-NPs through biosynthesis. The CaCO_3_-NPs, produced using ethanol, methanol, and an aqueous extract, demonstrated peak absorbances at 240 nm, 275 nm, and 250 nm, respectively. Figure 1 illustrates the calcium carbonate NPs synthesized using the methanol bark extract obtained from *A. altissima*.

#### 3.1.2 Fourier Transform Infra-Red spectroscopy

**FIGURE 2:**
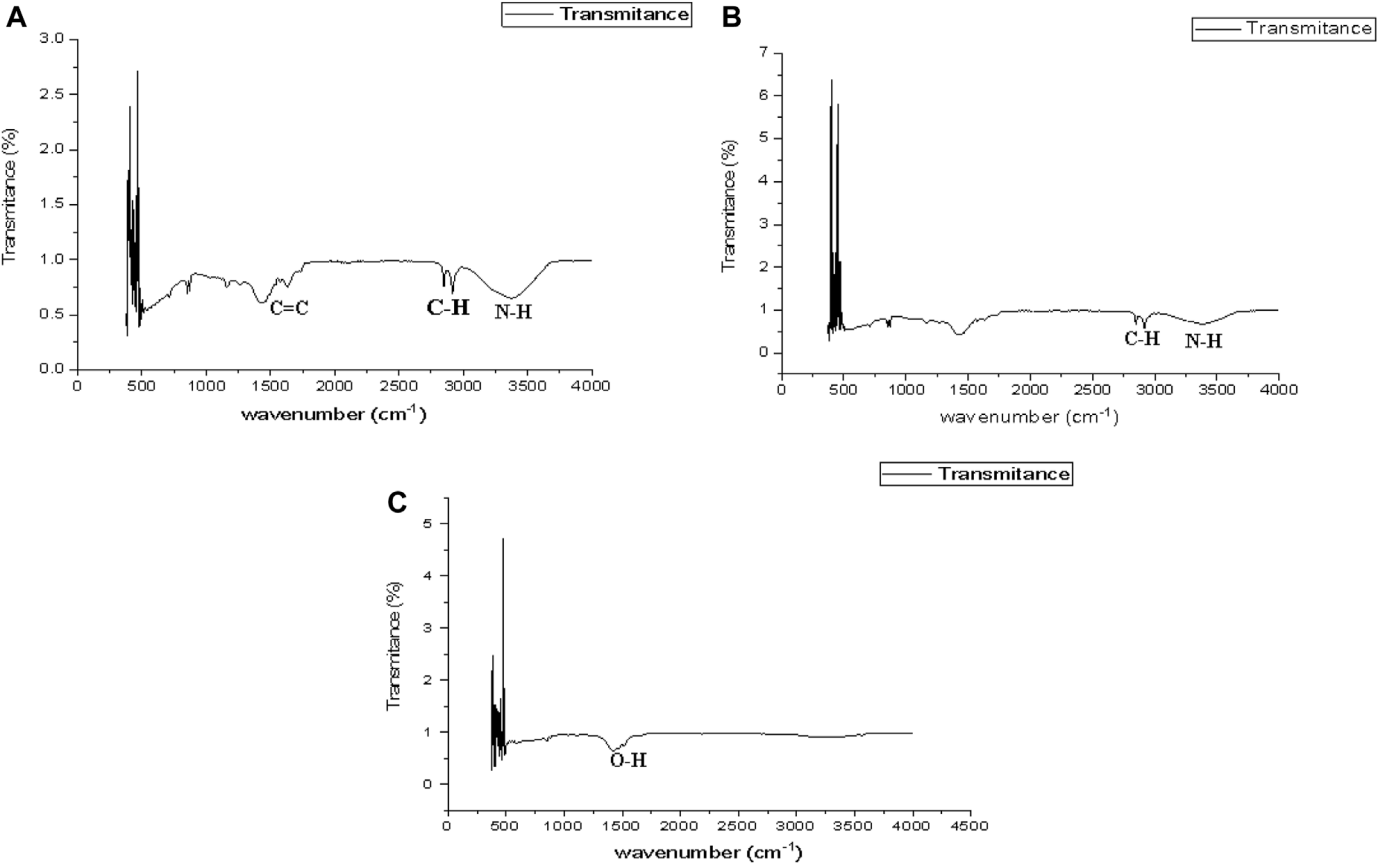
FTIR spectra of methanol **(A)**, ethanol **(B)**, and aqueous **(C)** CCNPs exhibited various peaks.

Bioactive compounds were analyzed using FTIR in order to identify their functional groups. Green-synthesized CaCO_3_-NPs displayed peaks of differing intensities (Figure 2). In the FTIR spectrum of calcium NPs, several peaks were observed at 2,800 cm^−1^, 2,900 cm^−1^, 3,400 cm^−1^, and 1,600 cm^−1^ indicating multiple stretches of functional groups. The carboxylic acid component is a member of the compound class O-H associated with the stretching band at 1600 cm^−1^ present in aqueous NPs. The C-H Stretching peak is shown in 2,800 cm^−1^ and 2,900 cm^−1^ and the N-H Stretching peak is present at 3,400 cm^−1^ in ethanol NPs while methanol NPs give C = C Stretching (1700 cm^−1^), C-H Stretching (2,800 and 2,900 cm^−1^), N-H Stretching **(**3,400 cm^−1^) as depicted in Figure 2.

#### 3.1.3 Scanning electron microscopy (SEM) and EDX

**FIGURE 3:**
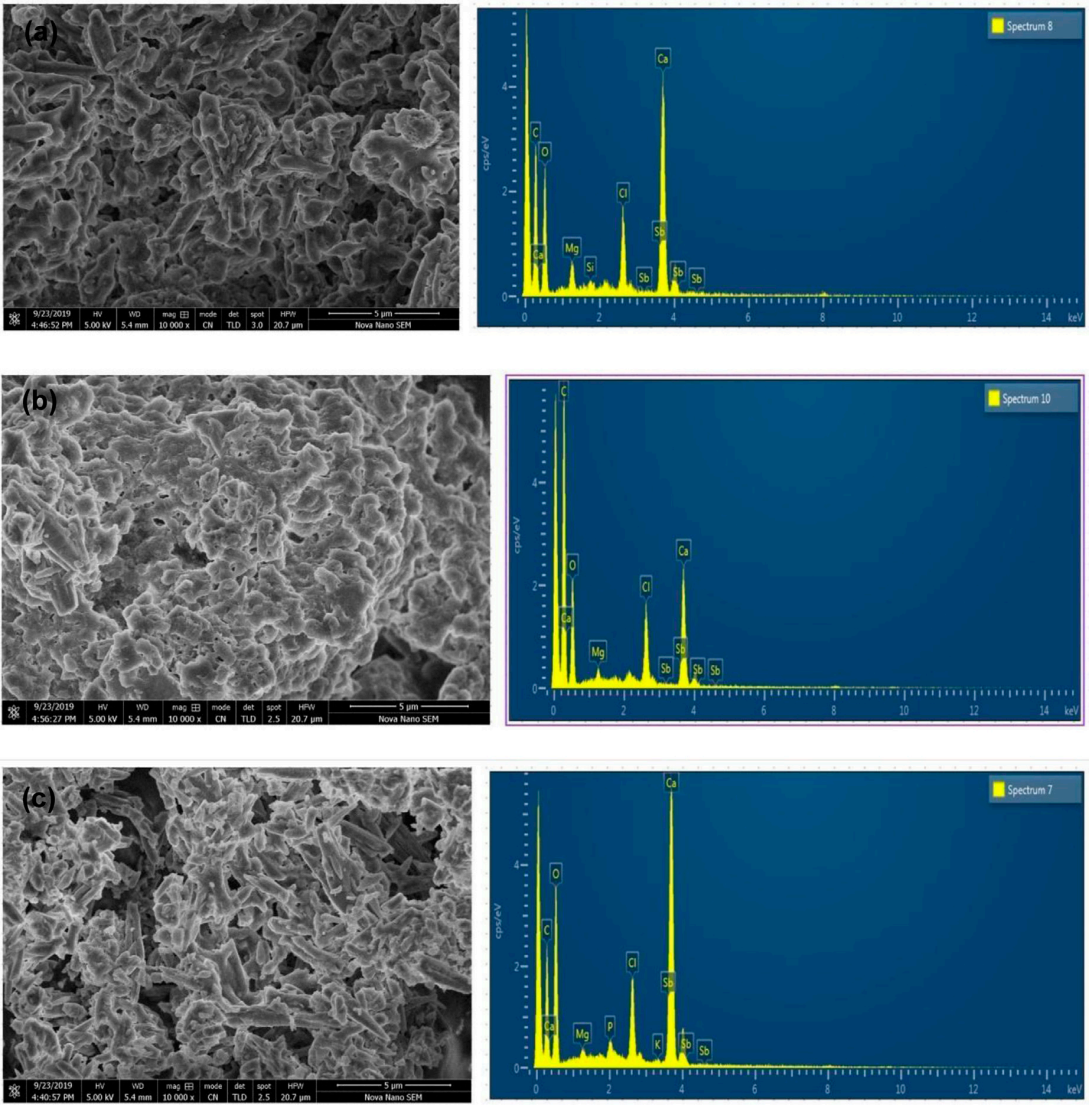
**(A)**, **(B)**, and **(C)** show the SEM and EDX of ethanol, Methanol, and Aqueous extracts, respectively, biosynthesized CaCO_3_-NPs derived from the bark of *Ailanthus altissima* at the 10,000 magnifications.

Micrographs were obtained using scanning electron microscopes at magnifications of almost 10,000. The determination of the particles’ solid core diameter was conducted via scanning electron micros-copy (SEM). All CaCO_3_ had an almost spherical (or globular) rod shape present in methanol, ethanol, and aqueous NPs which was shown in Figure 3. The EDX (Energy Dispersive *X*-ray) analysis has substantiated the existence of zinc oxide NPs, cultivated through the biosynthesis method. The EDX results indicate the presence of calcium, chlorine, and antimony, thereby providing compelling evidence of the high degree of purity attained in the synthesized CaCO_3_ NPs.

### 3.2 Biological activities

#### 3.2.1 Anti-oxidant activities

##### 3.2.1.1 Hydroxyl radical scavenging activity

**FIGURE 4:**
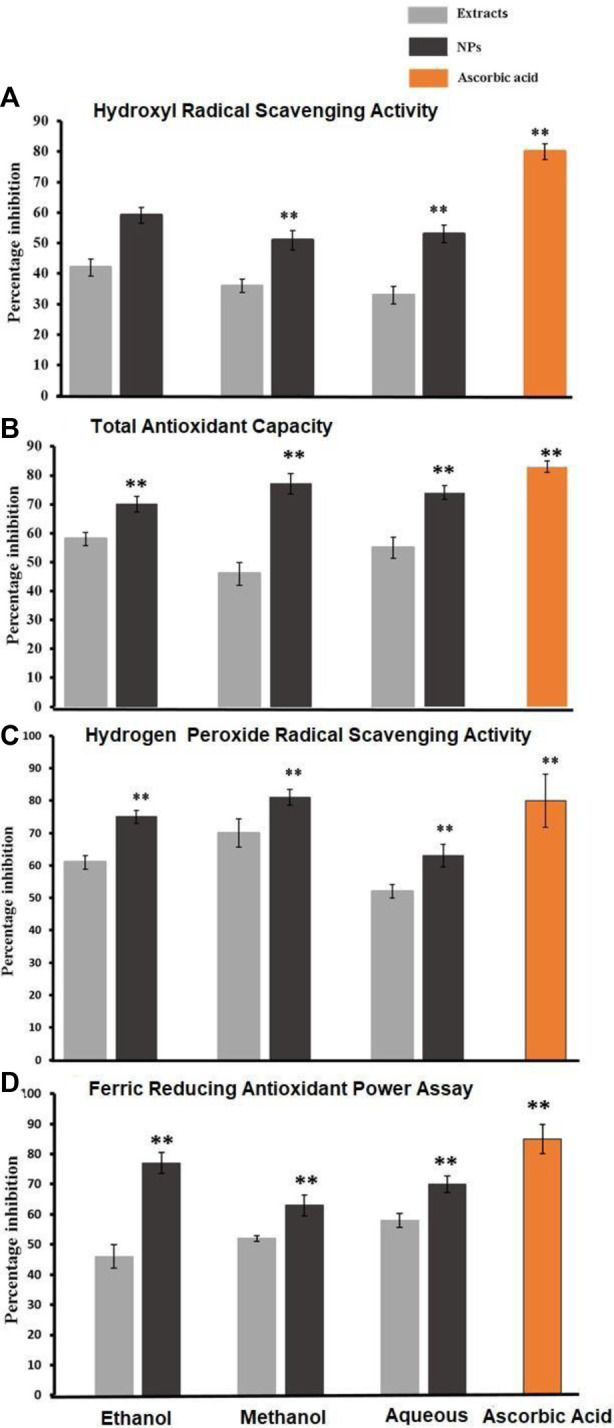
**(A)**, **(B)**, **(C)**, and **(D)** show hydroxyl radical scavenging activity, total antioxidant capacity, hydrogen peroxide radical scavenging activity, and ferric reducing antioxidant power, respectively, found in the crude extracts derived from the bark of *Ailanthus altissima* as well as the corresponding antioxidant capacity exhibited by the biosynthesized CaCO_3_. Data are expressed as mean ± S. D in triplicates all values are statistically significant *p* < 0.05.

The Smirnoff technique was employed for the assessment of hydroxyl radical scavenging activity in bark extracts and the corresponding CaCO_3_-NPs produced through biosynthesis. The quantification of inhibition percentages for both the extracts and their associated NPs were conducted through a paired sample *t*-test. As compared with their respective NPs, the plant extracts (ethanol, methanol, and aqueous) displayed a low inhibition level. Ascorbic acid exhibited 80% inhibition while the NPs showed 59% inhibition in ethanol CaCO_3_-NPs, 51% in methanol CaCO_3_, and 53% in aqueous CaCO_3_. Figure 4A below gives a percentage of inhibition.

##### 3.2.1.2 Phosphomolybdenum assay

The total antioxidant capacity of various bark extracts and their biosynthesized CaCO_3_ was assessed using the phosphomolybdenum assay. Paired sample *t*-tests were employed to analyze the percentage inhibition exhibited by different extracts and their corresponding NPs. The nanoparticles demon-strated a substantial percentage inhibition of 70%, whereas the simple ethanol bark extract displayed a comparatively lower inhibition rate of 58%. In the case of methanol NPs, the inhibitory effect observed was notably higher at 77%, while the inhibitory effect of the regular methanol bark extract was measured at 46%. Aqueous NPs exhibited a robust inhibitory effect of 74%, while the simple ethanol bark extract showed a slightly lower inhibition rate of 55% against free radicals. Ascorbic acid, used as a reference, displayed a remarkable 83% inhibition of free radicals, while the standard water bark extract exhibited a substantial 67% inhibition of free radicals. The data is graphically presented in Figure 4B.

##### 3.2.1.3 Hydrogen peroxide radical scavenging activity

The assessment of hydrogen peroxide radical scavenging activity was conducted on various bark extracts and their corresponding biosynthesized NPs. This evaluation employed a paired sample *t*-test to determine the statistical significance of the observed results. The percentage inhibition values recorded for the NPs were 75%, 81%, and 63%, while the ethanol extract exhibited a percentage inhibition of 61%. The methanol and aqueous extracts displayed inhibition percentages of 70% and 52%, respectively. In comparison, ascorbic acid exhibited a noteworthy inhibition rate of 80%, as depicted in Figure 4C.

##### 3.2.1.4 Ferric reducing antioxidants power assay

To determine the ferric-reducing activity of bark extracts and NPs produced through their biosynthesis, a paired sample *t*-test was used. As shown in Figure 4D, ascorbic acid showed 85% inhibition, while NPs showed 77%, 63%, and 70% inhibition, in contrast to simple ethanol extracts, which showed 46% inhibition, methanol extracts, which showed 63%, and aqueous extracts, which showed 70% inhibition.

#### 3.2.2 Anti-diabetic activity

##### 3.2.2.1 **α-**Amylase inhibition assay

**FIGURE 5:**
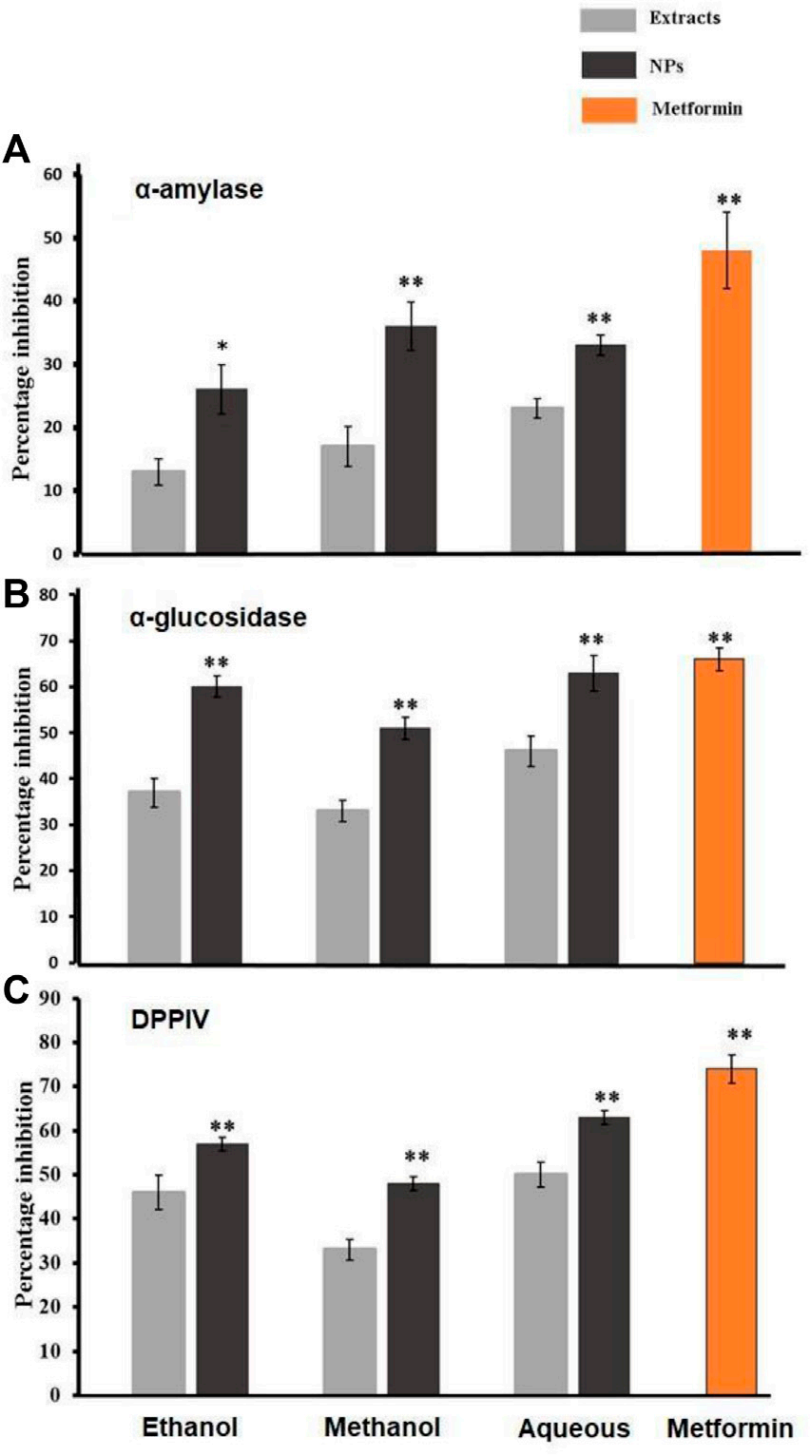
**(A)**, **(B)**, and **(C)** shows the percentage inhibition of α-amylase, α-glucosidase, and DPPIV found in the crude extracts derived from the bark of *Ailanthus altissima* as well as the corresponding antioxidant capacity exhibited by CO3. Data are expressed as mean ± S. D (*p* < 0.01 and *p* < 0.05) *n* = 5 the biosynthesized Ca.

The antidiabetic activity of bark extracts from *A. altissima* and their NPs was assessed by the α-Amylase inhibition assay. A paired sample *t*-test was utilized to evaluate the results. The antidiabetic activity of NPs and their respective NPs was assessed using an amylase inhibition assay. The results were then analyzed using a paired sample *t*-test. Inhibition percentages for NPs were 60%, 51%, and 63%, and those for ethanol, methanol, and aqueous extracts were 70%,52%, and 23% respectively. While metformin showed 66% inhibition, as shown in Figure 5A.

##### 3.2.2.2 α-Glucosidase inhibition activity

The assessment of the antidiabetic activity of NPs and their corresponding control NPs involved the utilization of an α-glucosidase inhibition assay. Subsequently, the obtained results were subjected to statistical evaluation using a paired sample *t*-test. Inhibition percentages for NPs were 60%, 51%, and 63%, and those for ethanol and methanol extracts were 70% and 52%, respectively. While metformin showed 66% inhibition, as shown in Figure 5B.

##### 3.2.2.3 Dipeptidyl peptidase IV inhibition assay

The evaluation of the potential antidiabetic properties of *A. altissima* bark extracts and their corresponding NPs was conducted through the Dipeptidyl peptidase IV (DPP-IV) inhibition assay. Subsequently, the obtained results were analyzed using the paired sample *t*-test to assess their statistical significance. Inhibition percentages for NPs were 57%, 48%, and 63%, and those for ethanol and methanol aqueous 50%, extracts were 46%, and 43%, respectively. While metformin showed 74% inhibition, as shown in Figure 5.

### 3.3 *In silico* analysis

#### 3.3.1 Docking analysis

**FIGURE 6:**
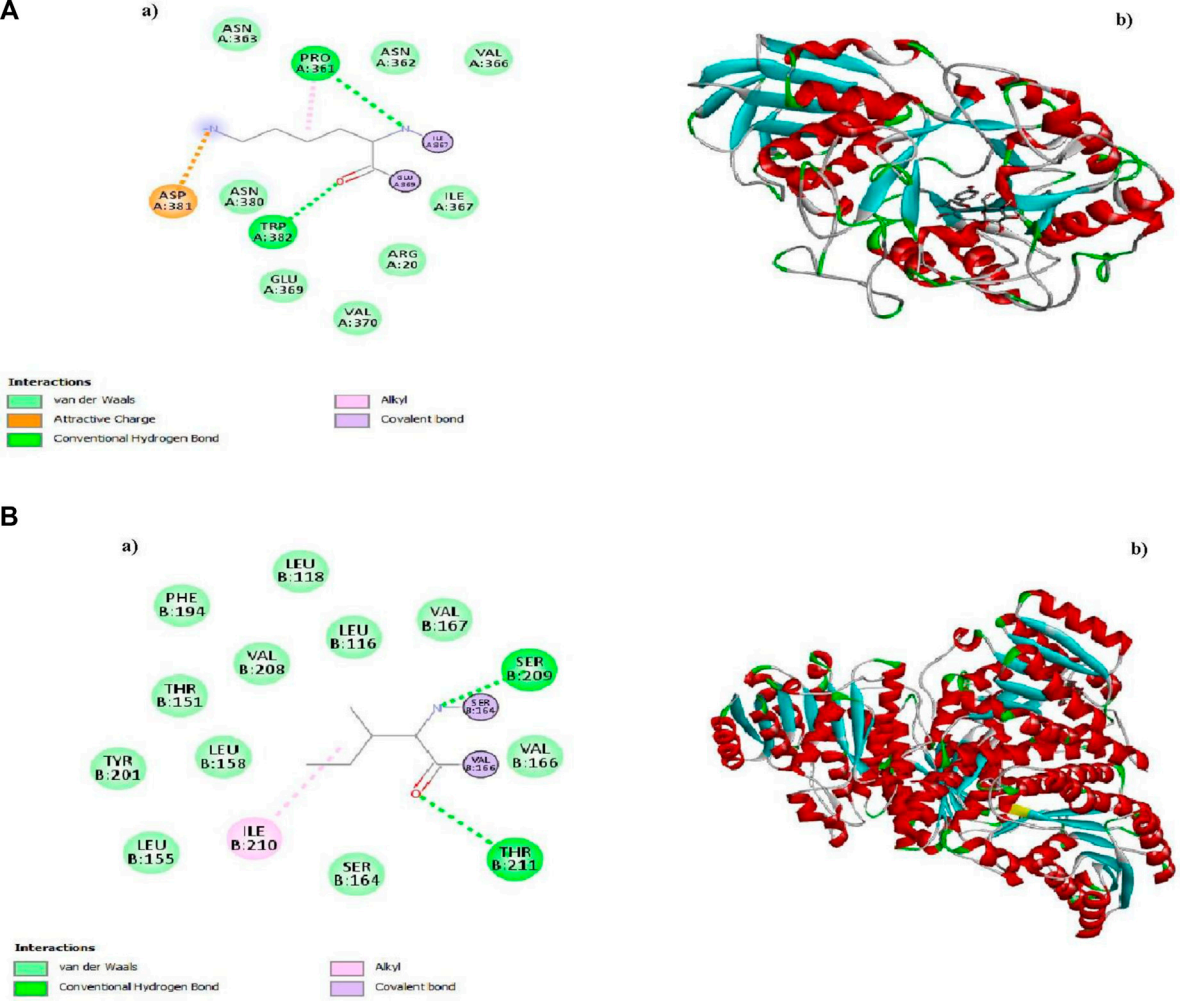
**A**(a) and **A**(b) 2D and 3D structures of Apigentrin, **B**(a) and **B**(b) 2D and 3D structures of Rutin.

Molecular Docking determines the binding energies, and the highest Apigetrin and Rutin show high binding energy scores against (1b2y) and −9 kJ/mol and (1xu7) −12 kJ/mol show more potential in type 2 diabetes cause protein which was shown in Table 1. The 3 days and 2days interactions are given below in Figure 6.

**TABLE 1:** Molecular docking of NPs against α-amylase and 1xu7.

Sr No	Name of compounds	PUB chem id	α-amylase (1b2y) (KJ/mol)	Tetrameric 11b- HSD (1xu7) (KJ/mol)
1	Apigetrm-Calcium car-bonate NPs (APTy-CaCO_3_)	5280704	−9	−11.1
2	Apigenin-Calcium car-bonate NPs (AP-CaCO_3_)	5280443	−8.9	−9.8
3	Catechin- Calcium car-bonate NPs (C-CaCO_3_)	9,064	−8.8	−9.8
**4**	Kaempferol- Calcium carbonate NPs (KMP-CaCO_3_)	5280863	−8.8	−9.8
5	Rutin- Calcium car-bonate NPs (RT-CaCO_3_)	5280805	−8.7	−12.1
6	Myricetin- Calciumcarbonate NPs (MR-CaCO_3_)	5281672	−8.7	−10.1
7	Fisetin- Calcium car-bonate NPs (FIS-CaCO_3_)	5281614	−8.7	−9.9
8	Quercetin- Calciumcarbonate NPs (QCT-CaCO_3_)	5280343	−8.6	−10
9	Luteolin- Calcium car-bonate NPs (LT-CaCO_3_)	5280445	−8.6	−10
10	Afzelin- Calcium car-bonate NPs (AFL-CaCO_3_)	5316673	−8.4	−11.8
11	Epicatechin- Calcium carbonate NPs (EC-CaCO_3_)	72276	−8.4	−9.8
12	Galangin- Calcium car-bonate NPs (GA-CaCO_3_)	5281616	−8.4	−9.8
13	Isoquerectin- Calcium carbonate NPs (IQ-CaCO_3_)	5280804	−8.3	−11.5
14	Cyanidin- Calcium car-bonate NPs (CY-CaCO_3_)	128861	−8.3	−9.8
15	Hesperetin- Calcium carbonate NPs(Hst-CaCO_3_)	72281	−8	−10
16	Astragalin- Calcium carbonate NPs (AG-CaCO_3_)	5282102	−7.9	−11.4
17	Isorhamnetin- Calcium carbonate NPs (IHT-CaCO_3_)	5281654	−7.8	−9.9
18	Chalcone- Calcium carbonate NPs (CH-CaCO_3_)	637760	−6.7	−8.1
19	Xanthoxylin- Calcium carbonate NPs (XTH-CaCO_3_)	66654	−5.4	−6.8
20	Genistein- Calcium carbonate NPs (GST-CaCO_3_)	5280961	−6.2	−6.9

#### 3.3.2 Imod simulation

##### 3.3.2.1 Molecular dynamic simulation

**FIGURE 7:**
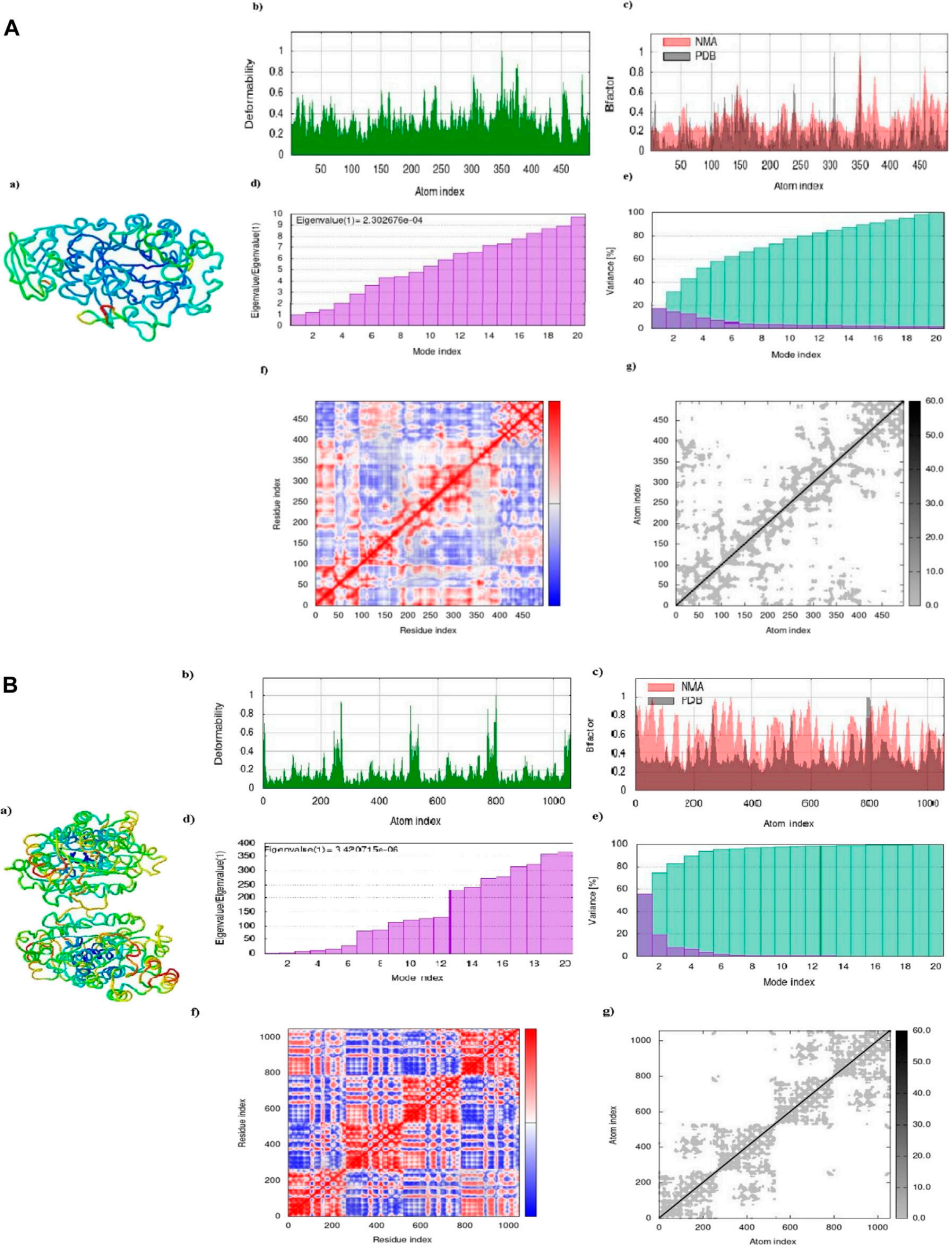
**(A)** Apigentrin. Complex showed a dynamic simulation of IMOD. **(B)** Rutin complex of dynamic simulation of Imod.

The results of the molecular dynamics study conducted on the Apigentrin and Rutin docked complex reveal notable characteristics. Specifically, the complex exhibits a significant degree of deformability, as evidenced by Figure 7. Furthermore, it is noteworthy that this complex possesses a remarkably low eigen value, measuring Apigentrin 2.302674e-04 and Rutin 3.420714e-06. This lower eigen value is indicative of heightened deformability within the complex, as depicted in Figure 12 days. Additionally, it serves as an indicator of the protein complex’s motion stiffness. Notably, the variance map illustrates a substantial accumulation of variances, surpassing individual variances, as depicted in below the Figure. The covariance and elastic network map also produced satisfactory results Figure 7.

#### 3.3.3 ADMET analysis

**FIGURE 8:**
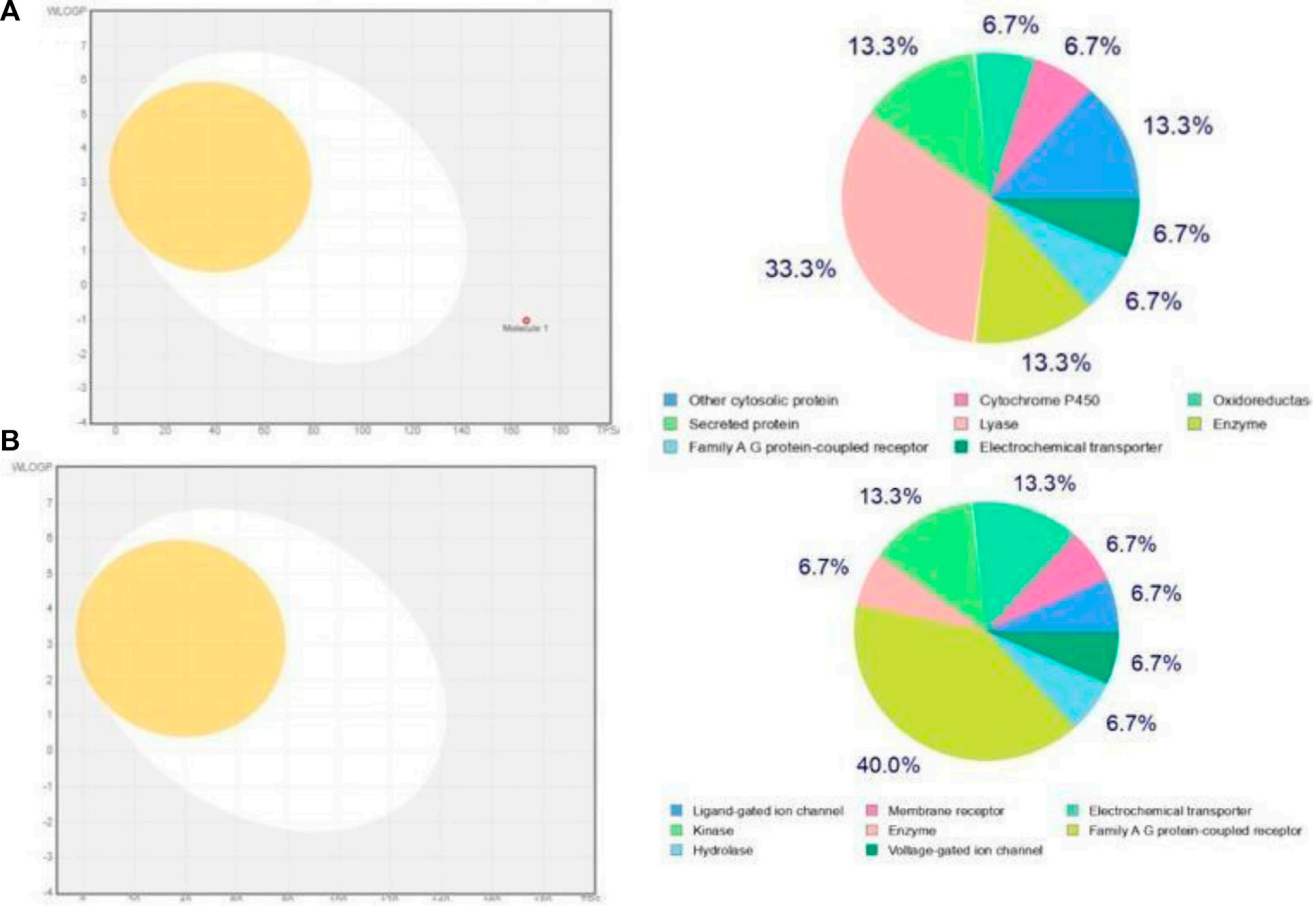
**(A)** Boiled-Egg models and bioavailability radar of Apigentrin and **(B)** Boiled-Egg models and bioavailability radar of Rutin.

A drug library (*n* = 673) underwent rigorous evaluation, including pharmacokinetics (PK), absorption, distribution, metabolism, excretion, and toxicity (ADMET) analysis, as well as pharmacodynamics and physicochemical assessment. Apigentrin and Rutin were selected based on their favorable properties: high gastrointestinal absorption, solubility, blood-brain barrier permeability, and compliance with Lipinski’s Rule of Five, Veber and Ghosh’s criteria, Egans’ principles, and Mugge’s rules. The Apigentrin water solubility is −1.58 while Rutin water solubility is −2.16. Additionally, compounds with good bioavailability, lead-like scores, and synthetic accessibility were prioritized. Following ADMET analysis, toxicological screening ensured the exclusion of compounds with potential toxicity or receptor binding. The boiled egg and bioavailability radar of predicted target prediction was shown in Figure 8.

## 4 Discussion

The biomolecules and bioreducers found in plants, such as enzymes, flavonoids, proteins, cofactors, and terpenoids provide an economical, versatile, and eco-friendly approach to fabricating metal NPs (Karimzadeh et al., 2020). Using medicinal plants for the biosynthesis of metal NPs has generated significant attention as an alternative to using hazardous physical and chemical methods (Anand et al., 2017). Different studies have shown the nutraceutical and pharmaceutical potential of NPs in which they interact with a high degree of sensitivity, specificity, and signaling capability at the molecular as well as cellular levels (Chandra et al., 2020). Because of its ready availability and slow biodegradation, CaCO_3_ finds utility in controlled drug delivery and the encapsulation of various drugs, including bioactive proteins, within the field of pharmaceutics. Additionally, CaCO_3_ is employed for bone substitution and serves as a drug carrier in the context of bone-related diseases or defects (Garg et al., 2021). In this present research, the CaCO_3_ were synthesized using various crude extracts (ethanol, methanol, aqueous) of *A. altissima.* The optimal method for structurally characterizing NPs (NPs) is ultraviolet-visible (UV-Vis) spectroscopy. NPs synthesized from *A. altissima* using ethanol, methanol, and aqueous solutions exhibit a pronounced UV absorption band, displaying absorption peaks at 240 nm, 275 nm, and 250 nm, as depicted in Figure 1. This phenomenon arises from their surface plasmon resonance. Moreover, the presence of these sharp peaks indicates that the particles possess nanoscale dimensions, characterized by a narrow particle size distribution. These findings align well with prior research, falling within the range of 233–356 nm, as reported in previous studies (Brittain, 2020). FTIR spectros-copy serves as a vital analytical tool for distinguishing various phases of both organic and inorganic compounds. Specifically, this technique proves especially valuable in the differentiation of CaCO_3_ phases, primarily attributed to disparities in the carbonate ions present. (Ramasamy et al., 2017). As illustrated in Figure 2, the FTIR spectra of CaCO_3_ reveal valuable insights into the biomolecule functional groups associated with *A. altissima*-synthesized CaCO_3_ across various wavenumber ranges. Notably, the ethanol-based NPs exhibit a distinctive C-H stretching peak at 2,800 cm^−1^ and 2,900 cm^−1^, along with an N-H stretching peak at 3,400 cm^−1^. Conversely, the methanol-based NPs display a C = C stretching signal at 1700 cm^−1^, alongside C-H stretching (2,800 and 2,900 cm^−1^) and N-H stretching (3,400 cm^−1^) as depicted in Figure 2. These findings serve to affirm the varied functional groups’ roles as reducing agents in the nanoparticle formation process, underscoring their potential significance in the synthesis mechanism (Krpetic et al., 2009). The morphological attributes of the calcium carbonate nanocrystal depicted in Figure 3 are representative scanning electron microscopy (SEM) micrographs of the samples under investigation. The SEM displayed in Figure 3 reveals that ethanol, methanol, and aqueous calcium carbonate NPs exhibit cube-like crystalline structures characteristic of calcite, which exhibit a high degree of stability when compared to the rod-like orthorhombic crystals observed in aragonite. These SEM-derived morphological findings are in concordance with those presented in a prior study (Widyastuti, 2017), thereby corroborating the observed crystalline forms. In humans, the buildup of free radicals is associated with many diseases. On the other hand, antioxidants can neutralize them and reduce their harm (Rahman et al., 2015). The *A. altissima* NPs can neutralize the hydroxyl radicals by reducing them. The hydroxyl radicals scavenging activity of NPs synthesized from bark ethanol (*t*1, 4 = −7.824 *p* < 0.01), methanol (t1, 4 = −6.91 *p* < 0.01) and aqueous (*t*1, 4 = −11.180 *p* < 0.01). The extract was significantly greater than the simple bark of the respective extract. The NPs showed a percentage of inhibition of 59% ethanol CaCO_3_, 51% methanol CaCO_3_, and 53% aqueous CaCO_3_ while ascorbic acid showed 80% of inhibition. Figure 4A shown a percentage of inhibition. Thus NPs were highly potential as compared to the plant extract. The phosphomolybdate method has become a conventional approach for the assessment of the overall antioxidant capacity of various extracts (Khan et al., 2012). The findings of the study demonstrate that biosynthesized CaCO_3_ derived from a 77% methanol extract of bark exhibits notably higher total antioxidant capacity in comparison to NPs produced from aqueous (74%) and ethanol (70%) extracts. This implies that CaCO_3_ synthesized using methanol is particularly effective at scavenging free radicals, while those produced with acetone display a comparatively reduced capacity to neutralize free radicals (Nasim, 2022). The nanoparticle-based treatments exhibited substantial inhibitory effects, with percentages of inhibition recorded at 75%, 81%, and 63%. Conversely, the simple ethanol extract demonstrated a slightly lower inhibition rate of 61%, while the methanol and aqueous extracts displayed inhibition rates of 70% and 52%, respectively. In comparison, ascorbic acid exhibited the highest inhibition percentage at 80%, as illustrated in the accompanying Figure 4B. The scavenging effects of different nanoparticle extracts derived from *A. altissima* were observed in terms of their inhibition of hydrogen peroxide, with percentages of inhibition recorded at 75%, 81%, and 63% for various extracts. In contrast, a simple ethanol extract exhibited 61% inhibition, while methanol and aqueous extracts showed inhibition percentages of 70% and 52%, respectively. In comparison, ascorbic acid displayed a notable 80% inhibition, as illustrated in the accompanying Figure 4C. These findings indicate the high efficacy of NPs against the respective plant extracts. The FRAP assay, employed to assess the reducing potential of antioxidants by reacting with a ferric tripyridyltriazine (Fe3+ -TPTZ) complex and yielding a colored ferrous tripyridyltriazine (Fe2+ -TPTZ), underscores the process of free radical chain disruption through hydrogen atom donation. A paired sample *t*-test was performed to determine the ferric-reducing activity of bark extracts and NPs produced through their biosynthesis. Ascorbic acid showed 85% inhibition as shown in the figure while NPs showed 77%, 63%, and 70% inhibition, as opposed to simple ethanol extract, which showed 46% inhibition in methanol extract, which showed 63%, and aqueous extract, which showed 70% inhibition. The antioxidant assay of calcium carbonate are novelty base which are highly effective as compared to the plant extract shown in Figure 4D. The enzyme alpha-amylase, primarily found in saliva and pancreatic juice, plays a pivotal role in the hydrolysis of polysaccharides containing 1, 4-glucan, such as starch. The inhibition of this enzyme can serve as an effective means to prevent elevated blood glucose levels (Afzal et al., 2023). Inhibition percentages for NPs were 60%, 51%, and 63%, and those for ethanol, methanol, and aqueous extracts were 70%,52%, and 23% respectively. While metformin showed 66% inhibition, as shown in Figure 5A.

The findings of the study demonstrate that CaCO_3_ synthesized using an aqueous extract derived from *A. altissima* bark exhibited greater antidiabetic activity when compared to CaCO_3_ produced from ethanol, methanol, and aqueous extracts of *A. altissima* bark. Several prior investigations have proposed the efficacy of plant extracts combined with NPs as inhibitors of α-glucosidase, thus underscoring the promising potential of these extracts in the management of hyperglycemia (Bhatia et al., 2019). Inhibition percentages for NPs were 60%, 51%, and 63%, and those for ethanol and methanol extracts were 70% and 52%, respectively. While metformin showed 66% inhibition, as shown in Figure 5B. Inhibiting the α-glucosidase enzyme presents a valuable strategy for effectively managing Type II diabetes. This approach capitalizes on the role of incretin hormones, specifically glucagon-like peptide-1 (GLP-1) and glucose-dependent insulinotropic polypeptide (GIP), in stimulating insulin secretion by approximately 50%–60% in a glucose-dependent fashion (Gao et al., 2020). In our current study, the NPs were 57%, 48%, and 63%, and those for ethanol and methanol aqueous were 50%, and extracts were 46%, and 43%, respectively. While metformin showed 74% inhibition, as shown in Figure 5C. The current results from previous repoterted (Shukla et al., 2019). The findings suggest that CaCO_3_ synthesized using an aqueous extract derived from *A. altissima* bark demonstrated superior antidiabetic properties when compared to calcium carbonate NPs produced from ethanol, methanol, and aqueous extracts of the same *A. altissima* bark.

It has been found that 11β-hydroxysteroid dehydrogenase type I (11β HSD 1) is more active in lean individuals with type 2 diabetes (Das et al., 2022). Additionally, human pancreatic alpha-amylase (PDB id 1b2y) was identified as a contributor to increased postprandial glucose levels in diabetic patients. These findings suggest potential targets for diabetes research and management (Poovitha and Parani, 2016). In the current study, insilco analysis of type-2 diabetes shows the NPs are effective against type protein (1b2y) and (1xu7). In previous studies, 20 flavonoids of *A. altissima* had been identified. By using chem draw, NPs were drawn, and by using Discovery Studio, a 3-dimensional structure was prepared.

During the preparatory phase of establishing the binding site, we opted for the PDB format for the drugusing the Pyrx software to conduct virtual screening. Subsequently, molecular docking was employed as a methodology to assess binding affinities and gain insights into the prospective interactions between proteins and ligands. While Apigetrin and Rutin compounds give a high binding score as compared to the other bioactive components. MD simulation further ratified the stability of the docked complexes between the phytochemicals and protein through strong hydrogen bonding (Rao and Hariprasad, 2021). Our In Silico data also indicated that Apigetrin and Rutin might block human TLR4, which could be useful in type 2 diabetes protein Tetrameric 11B-HSD1I and human pancreatic alpha-amylase those our result are similar with previous literature [43]. Based on the assessment of ADME (Absorption, Distribution, Metabolism, and Excretion) characteristics and drug-like properties of the aforementioned molecules which was shown in Figure 8, it is evident that they exhibit a high level of bioavailability within the gastrointestinal tract. However, they do not display significant permeability across the blood-brain barrier (BBB). The determination of a molecule’s drug-likeness according to the bioavailability radar takes into consideration six key physicochemical properties: saturation, polarity, flexibility, size, lipophilicity, and solubility. Notably, *in silico* analysis has indicated the effectiveness of our NPs against type 2 diabetes, underscoring their potential therapeutic value in this context.

## 5 Conclusion

In conclusion, we have reported for the first time that the green synthesis of CaCO_3_ using various extracts of *A. altissima.* Biomolecules present in *A. altissima* extracts acted as both reducing and stabilizing agents, thereby eluding the requirement for external reducing agents. The effect of reducing agents on morphological features was observed in SEM micrographs and EDX while FTIR depicted the functional group. The synthesized CaCO_3_-NPs exhibited promising potential in anti-oxidant and anti-diabetic assays. The drug-likeness and ADMET studies of CaCO_3_-NPs revealed good medicinal and chemotherapeutic profile in agreement with all the assessed parameters of being drug. The molecular docking affinity scores, and MD-simulation stability assessments, as well as drug-likeness profiling and ADMET study evaluations, collectively suggest that CaCO_3_-NPs holds potential as a promising anti-oxidant and antidiabetic therapeutic candidate. The therapeutic potential profile of CaCO_3_-NPs observed *in vitro* aligns closely with the predictions generated through *in silico* analysis. It is suggested that green synthesized CaCO_3_-NPs could be used for the treatment of various ailments and/or metabolic diseases, and opening a door for a new range of bio-inspired synthetic nano-medicinal agents.

## Data Availability

All the data of this study is contained in this manuscript. More data related to this study can be accessed upon a reasonable request to the corresponding author NS at noreen.samad@bzu.edu.pk.
