# Supplementary material for: A novel approach to assessing the antioxidant and anti-diabetic potential of synthesized calcium carbonate nanoparticles using various extracts of Ailanthus altissima
Source: Front Chem. 2024 Jun 3;12:1345950. doi: 10.3389/fchem.2024.1345950 (PMC11182424; doi:10.3389/fchem.2024.1345950)
Supplement: Supplementary file 1 [file DataSheet1.PDF]

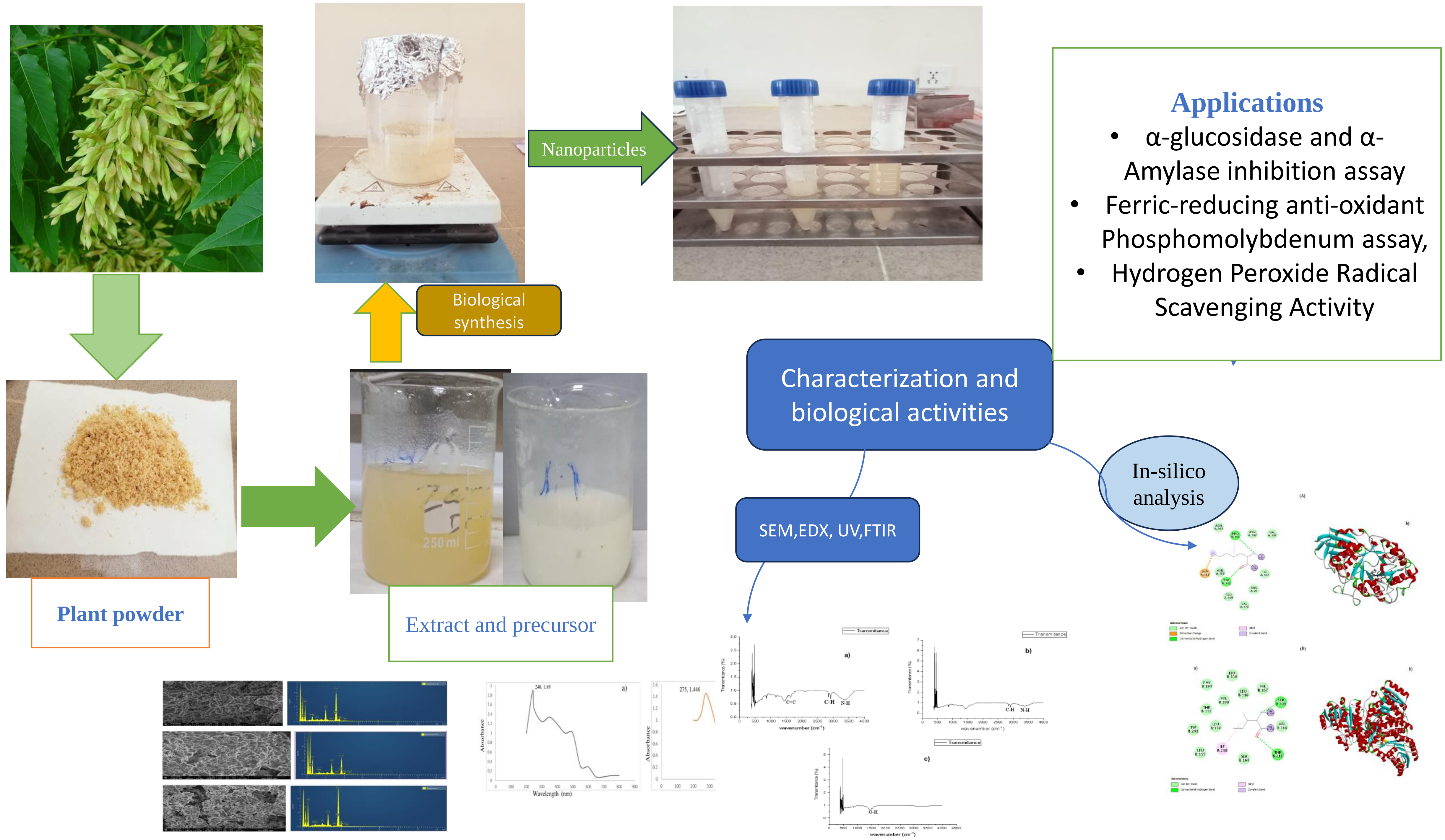

Nanoparticles
Biological synthesis
Plant powder
Extract and precursor
Applications
α-glucosidase and α-Amylase inhibition assay
Ferric-reducing anti-oxidant Phosphomolybdenum assay,
Hydrogen Peroxide Radical Scavenging Activity
Characterization and biological activities
In-silico analysis
SEM,EDX, UV,FTIR
